# Sex-specific effects of perinatal dioxin exposure on eating behavior in 3-year-old Vietnamese children

**DOI:** 10.1186/s12887-018-1171-2

**Published:** 2018-07-05

**Authors:** Anh Thi Nguyet Nguyen, Muneko Nishijo, Tai The Pham, Nghi Ngoc Tran, Anh Hai Tran, Luong Van Hoang, Hitomi Boda, Yuko Morikawa, Yoshikazu Nishino, Hisao Nishijo

**Affiliations:** 1 Department of Public Health and Epidemiology, Kanazawa Medical University, 1-1, Daigaku, Uchinada, Ishikawa 920-0293 Japan; 2 Biomedical and Pharmaceutical Research Center, Vietnam Military Medical University, Ha Noi, Vietnam; 3 School of Nursing, Kanazawa Medical University, 1-1 Daigaku, Uchinada, Ishikawa 920-0293 Japan; 4 System Emotional Science, Graduate School of Medicine and Pharmaceutical Sciences, University of Toyama, 2630 Sugitani, Toyama, 930-0194 Japan

**Keywords:** Dioxin, Breast milk, Perinatal exposure, Children, Eating behavior, Vietnam

## Abstract

**Background:**

We previously reported that perinatal dioxin exposure increased autistic traits in children living in dioxin-contaminated areas of Vietnam. In the present study, we investigated the impact of dioxin exposure on children’s eating behavior, which is often altered in those with developmental disorders.

**Methods:**

A total of 185 mother-and-child pairs previously enrolled in a birth cohort in dioxin-contaminated areas participated in this survey, conducted when the children reached 3 years of age. Perinatal dioxin exposure levels in the children were estimated using dioxin levels in maternal breast milk after birth. Mothers were interviewed using the Children’s Eating Behaviour Questionnaire (CEBQ). A multiple linear regression model was used to analyze the association between dioxin exposure and CEBQ scores, after controlling for covariates such as location, parity, maternal age, maternal education, maternal body mass index, family income, children’s gestational age at delivery, and children’s age at the time of the survey. A general linear model was used to analyze the effects of sex and dioxin exposure on CEBQ scores.

**Results:**

There was no significant association between most dioxin congeners or toxic equivalencies of polychlorinated dibenzo-*p*-dioxins/furans (TEQ-PCDDs/Fs) and CEBQ scores in boys, although significant associations between some eating behavior sub-scores and 1,2,3,4,6,7,8,9-octachlorodibenzofuran were observed. In girls, there was a significant inverse association between levels of TEQ-PCDFs and enjoyment of food scores and between levels of TEQ-PCDFs and TEQ-PCDDs/Fs and desire to drink scores. Two pentachlorodibenzofuran congeners and 1,2,3,6,7,8-hexachlorodibenzofuran were associated with a decreased enjoyment of food score, and seven PCDF congeners were associated with a decreased desire to drink score. The adjusted mean enjoyment of food score was significantly lower in children of both sexes exposed to high levels of TEQ-PCDFs. There was, however, a significant interaction between sex and TEQ-PCDF exposure in their effect on desire to drink scores, especially in girls.

**Conclusions:**

Perinatal exposure to dioxin can influence eating behavior in children and particularly in girls. A longer follow-up study would be required to assess whether emotional development that affects eating styles and behaviors is related to dioxin exposure.

**Electronic supplementary material:**

The online version of this article (10.1186/s12887-018-1171-2) contains supplementary material, which is available to authorized users.

## Background

Dioxin contamination in Vietnam has had a long-term impact on the environment and human health, particularly in hotspots of high contamination [1–4]. This has mainly been related to the use of herbicides, especially Agent Orange, which contains 2,3,7,8-tetrachlorodibenzo-*p*-dioxin (TCDD) during the Vietnam War. We found that dioxin levels in maternal breast milk in contamination hotspots in southern Vietnam were 3–4 times the levels in unsprayed areas of northern Vietnam, even though four decades had passed [5]. As breast milk reflects the maternal body burden of dioxin, we carried out various studies on the health effects of dioxin exposure in infants using dioxin levels in maternal breast milk as a marker of perinatal exposure. We found that perinatal dioxin exposure was associated with reduced growth during the first 4 months of life, particularly in boys [6], lower cognitive and fine motor skill scores in infants aged 4 months [7], and lower social emotional scores in toddlers aged 1 year [8].

When our cohort reached 3 years of age, we performed a health impact survey to determine whether there was an association between perinatal dioxin exposure and autistic traits measured using the Autism Spectrum Rating Scales (ASRS), and found that perinatal TCDD exposure increased social emotional difficulties in these children [9]. We also investigated the longitudinal effects of perinatal dioxin exposure on growth and neurodevelopment in the children during the first 3 years of life, and reported that increased dioxin exposure was associated with sex-specific effects on growth and neurodevelopment. We found that exposure decreased various body measurements in boys, increased head and abdominal circumferences in girls, and decreased neurodevelopmental motor and expressive language scores in boys [10]. These findings highlight the need to investigate sex-specific effects of perinatal dioxin exposure on children’s eating behavior, which may lead to differences in size, particularly in children with poor neurodevelopment. In clinical studies, eating behavior has been linked to problems in social behavior in children with neurodevelopmental disorders such as autism spectrum disorder [11, 12], which are more prevalent in boys.

Animal studies have reported that dioxin exposure affected brain regions in the limbic system such as the hypothalamus, nucleus accumbens, amygdala, and orbitofrontal cortex [13–16] that are involved in controlling appetite and food rewards [17, 18], and altered taste preference in female rats exposed to dioxin perinatally [19]. Based on these findings, we hypothesized that dioxin may alter eating behaviors in children perinatally exposed to dioxin by affecting these brain regions. In the present study, we investigated whether dioxin exposure affected the eating behaviors of Vietnamese children, and whether such an effect was sex-specific.

## Methods

### Study subjects

The study was conducted in the Thanh Khe and Son Tra districts of Da Nang City near Da Nang airbase, where extremely high dioxin levels have been recorded in soil and sediment samples collected in 2006 (858–361,000 pg/g dry weight of TCDD in soil and 674–8580 pg/g dry weight in sediment from a nearby lake and from the airbase’s drainage system) [4]. The Vietnamese and US governments have been carrying out soil remediation activities at the Da Nang airbase since 2012 [20], but environmental contamination remains high.

In 2008–9, a total of 241 mother-and-newborn pairs (159 pairs in Thanh Khe and 82 in Son Tra) were recruited in district hospitals. These pairs were selected based on the following criteria: i) Mothers resided in Thanh Khe or Son Tra at least during their pregnancy; ii) babies were born at term; iii) mothers had no birth complications. Breast milk samples (20 mL) were collected from these mothers 1 month postpartum with the assistance of midwives and were stored in sterile polyethylene containers at − 4 °C at local health centers.

A total of 198 mother-child pairs (82.2%) participated in the survey when the children reached 3 years of age. Forty-one other pairs were lost to follow-up owing to relocation or failure to appear at the examination; two infants died in their first postnatal month. There were no significant differences in the characteristics of mothers and children or dioxin levels in breast milk between study participants and the 43 pairs lost to follow-up. Some demographic information was missing for 13 of the mothers, so the final sample for analysis included 185 mother-child pairs, representing 76.8% of the original sample.

At the follow-up surveys at 4 months, 1 year, and 3 years of age, neurodevelopmental measurements were obtained using the Bayley Scales of Infant and Toddler Development, Third Edition (NCS Pearson, Inc., Bloomington, MN, USA) to assess infant development in the areas of cognition, language, and motor functions. The findings were reported in our previous studies [6–9]. At the 3-year survey, full-length parent rating forms of the Autism Spectrum Rating Scales (ASRS; MHS, Inc., North Tonawanda, NY, USA) and the Children’s Eating Behaviour Questionnaire (CEBQ) [21] were used to measure behaviors associated with autism spectrum disorder and eating behavior, respectively. We previously reported an association between dioxin exposure and ASRS [9].

### Dioxin measurement in breast milk samples

Breast milk samples were frozen after collection and transported to the High Technology Center, Kanazawa Medical University, Japan to analyze concentrations of 17 2,3,7,8-substituted dioxin congeners. After extracting fat from 10 mL of sample and a series of purifying operations, measurement was performed using a gas chromatograph (HP-6980; Hewlett-Packard, Palo Alto, CA, USA) equipped with a high-resolution mass spectrometer (MStation-700, JEOL, Tokyo, Japan). The established analytical method has been described in detail previously [5]. Concentrations of the 17 congeners of polychlorinated dibenzo-*p*-dioxins/furans (PCDDs/Fs) were lipid-base calculated. Toxic equivalency factors (TEFs) for each congener were referenced from the World Health Organization 2005-TEF list [22]. Toxic equivalencies (TEQs) of polychlorinated dibenzo-*p*-dioxins (PCDDs), polychlorinated dibenzofurans (PCDFs), and PCDDs/Fs were calculated by multiplying each congener concentration by its TEF and summing the values. The values of the concentrations that were below the limit of detection were set to equal half of the limit of detection for the analysis. Table 1 lists the concentrations of each congener in breast milk samples, stratified by the sex of the child.

**Table 1:** Dioxin concentrations in maternal breast milk stratified by child’s sex

		Samples with	Boys			Girls		
	LOD	below LOD	(*N* = 106)			(*N* = 79)		
	(ppt)	N (%)	Median	GM	GSD	Median	GM	GSD
PCDD congeners (pg/g lipid)								
2,3,7,8-TetraCDD	0.004	11 (5.9)	1.5	1.3	2.1	1.7	1.4	2.6
1,2,3,7,8-PentaCDD	0.011	0	4.2	4.3	1.6	4.5	4.1	1.7
1,2,3,4,7,8-HexaCDD	0.004	0	2.2	2.3	1.6	2.4	2.3	1.7
1,2,3,6,7,8-HexaCDD	0.01	0	8.3	8.2	1.6	8.4	8.2	1.8
1,2,3,7,8,9-HexaCDD	0.008	0	2.5	2.6	1.6	2.7	2.7	1.7
1,2,3,4,6,7,8-HeptaCDD	0.008	0	12.1	12.0	1.5	11.4	12.1	1.6
OctaCDD	0.008	0	63.9	67.7	1.5	68.1	67.7	1.6
PCDF congeners (pg/g lipid)								
2,3,7,8-TetraCDF	0.005	13 (7.0)	0.5	0.5	2.0	0.6	0.5	2.2
1,2,3,7,8-PentaCDF	0.006	0	1.2	1.2	1.7	1.3	1.2	2.1
2,3,4,7,8-PentaCDF	0.01	0	7.2	7.1	1.5	8.1	7.2	1.7
1,2,3,4,7,8-HexaCDF	0.008	0	16.5	16.7	1.6	19.9	18.0	1.9
1,2,3,6,7,8-HexaCDF	0.003	0	10.8	10.2	1.6	12.0	11.1	1.9
1,2,3,7,8,9-HexaCDF	0.004	12 (13.5)	0.2	0.2	2.2	0.3	0.3	2.7
2,3,4,6,7,8-HexaCDF	0.006	0	1.3	1.2	1.5	1.5	1.4	1.9
1,2,3,4,6,7,8-HeptaCDF	0.01	0	10.8	11.6	1.6	13.5	12.9	2.0
1,2,3,4,7,8,9-HeptaCDF	0.004	3 (1.6)	1.2	1.1	1.9	1.6	1.2	2.7
OctaCDF	0.013	47 (25.4)	0.5	0.6	2.4	0.5	0.6	2.6
TEQs (pg-TEQ/g lipid)								
TEQ-PCDDs			7.4	7.3	1.6	8.0	7.3	1.8
TEQ-PCDFs			5.2	5.3	1.5	5.7	5.6	1.8
TEQ-PCDDs/Fs			12.5	12.6	1.5	14.2	13.0	1.7

*CDD* chlorinated dibenzo-*p*-dioxin, *CDF* chlorinated dibenzofuran, *GM* geometrical mean, *GSD* geometrical standard deviation, *LOD* limit of detection, *PCDD* polychlorinated dibenzo-*p*-dioxin, *PCDF* polychlorinated dibenzofuran, *ppt* parts per trillion, *TEQ* toxic equivalency

Lower TEQ levels of dioxin-like polychlorinated biphenyls (dl-PCBs) measured in the present study were compared with levels in samples collected in unsprayed areas in northern Vietnam, although dl-PCB levels were measured in only four samples [6]. Those findings suggested that TEQ-PCDDs/Fs can be used as an indicator of dioxin toxicity in the Da Nang sampling area because of the lower contribution of dl-PCBs to the total TEQ.

### Questionnaire survey

Structured questionnaires were used to obtain information related to childbirth and the growth and neurodevelopment of children (gestational weeks at birth and birth weight) and to maternal demographics at the 4-month survey. Maternal height and weight were obtained from medical records in the hospitals’ obstetrics departments. Table 2 lists the characteristics of the participants, stratified by the sex of the child. All children were born at full term, and breast milk served as the only or main food supply of all infants for the first 4 months. The children’s age at the time of the present survey was 34–40 months.

**Table 2:** Characteristics of study participants and Children’s Eating Behaviour Questionnaire (CEBQ) scores stratified by sex

		Boys		Girls	
		(N = 106)		(N = 79)	
	Characteristics, CEBQ scores	Mean, N (%)	SD	Mean, N (%)	SD
Mothers	Age (years)	27.9	5.7	28.4	6.6
	Primiparae (%)	30 (28.3)		25 (32.9)	
	Weight during pregnancy (kg)	58.4	6.3	56.7	7.1
	Height (cm)	154.4	4.9	154.0	5.3
	BMI	24.5	2.6	23.9	2.5
	Family income (VND)	2986	1489	3012	1717
Children	Age at the survey (months)	36.3	1.5	36.8	1.6
	Birth weight (g)	3280	384	3187	352
	Gestational weeks at birth	39.6	0.8	39.5	0.8
	Weight at the survey (kg)	14.1	1.9	13.4	1.9
	Height at the survey (cm)	93.2	3.2	91.9	3.6
	BMI at the survey	16.2	1.5	15.8	1.6
	*CEBQ scores*				
	Satiety Responsiveness (SR)	2.3	0.7	2.5	0.7
	Slowness in Eating (SE)	2.7	0.8	3.0	0.9
	Fussiness (FU)	3.1	0.4	3.2	0.4
	Food Responsiveness (FR)	2.6	1.0	2.9	1.0
	Enjoyment of Food (EF)	4.1	0.8	4.0	0.8
	Desire to Drink (DD)	3.3	1.3	3.4	1.2
	Emotional of Undereating (EU)	3.2	0.9	3.5	0.9
	Emotional of Overeating (EO)	2.0	0.6	2.3	0.8
	Food Approach (FAPP)	3.0	0.7	3.1	0.6
	Food Advoident (FAVD)	2.8	0.4	3.1	0.4

The food approach score was calculated from the food responsiveness, enjoyment of food, desire to drink, and emotional over-eating scores

The food avoidance score was calculated from the satiety responsiveness, slowness in eating, fussiness, and emotional under-eating scores

*N* number of subjects, *SD* standard deviation, *BMI* body mass index, *VND* Vietnam Dong

Mothers were asked about the children’s eating behavior in face-to-face interviews using the CEBQ, a multi-dimensional, parent-reported questionnaire [21]. This scale was applied after it had been translated from the original English into Vietnamese and then back-translated into English for validation. The CEBQ comprises 35 questions, scored on a 1–4 scale. The questions are divided into eight subscales, as follows, and the mean of each subscale was calculated: Satiety responsiveness, slowness in eating, fussiness, food responsiveness, enjoyment of food, desire to drink, emotional under-eating, and emotional over-eating. We summed the food responsiveness, enjoyment of food, desire to drink, and emotional over-eating scores to calculate the food approach (FAPP) score, and summed the satiety responsiveness, slowness in eating, fussiness, and emotional under-eating scores to calculate the food avoidance (FAVD) score. Significant correlations were observed between each of the subscales and the score under which it was categorized, suggesting internal CEBQ consistency among the present subjects. Table 2 lists the means and standard deviation of the eight subscales and the FAPP and FAVD scores, stratified by sex. In general, the CEBQ scores in the present study were higher than those reported in two European studies [23, 24]. Because of different distributions, we transformed the scores into standardized z-scores prior to analysis, which allowed calculation of the probability of a score occurring within our normal distribution.

Informed consent to participate in the present study was obtained from all mothers. The Institutional Ethics Boards of Medical and Epidemiological Studies at Kanazawa Medical University and Vietnam Military Medical University approved the study design. The Department of Health and Prevention of Diseases of Da Nang City government reviewed and approved the informed consent process.

### Statistical analysis

Statistical analysis was performed using SPSS ver. 11.0 for Windows (IBM SPSS, Armonk, NY, USA). Concentrations of PCDDs and PCDFs congeners and TEQ levels of PCDDs, PCDFs, and PCDDs/Fs in breast milk were used for analysis after base-10 logarithmical transformation to improve normality. A linear regression model was used to analyze the association between each CEBQ score (a response variable) and congener concentration and TEQ in breast milk (a predictor variable) after adjusting for the following covariates: Location (Thanh Khe or Son Tra), maternal age, parity, body mass index (BMI) during pregnancy, maternal education, family income, gestational weeks to delivery, and children’s age at the time of the survey. Regression analysis was stratified by sex because we previously found that the effects of dioxin exposure on growth and neurodevelopment differed between boys and girls in this cohort [5–10]. Next, a general linear model was used to analyze the effects of sex and dioxin levels on CEBQ scores by two-way ANCOVA after adjusting for the same covariates.

## Results

Tables 3 and 4 list the values calculated for the association between dioxin congener levels in breast milk and CEBQ scores in boys and girls aged 3 years, respectively. In boys, no significant association was found for TCDD or TEQ-PCDDs/Fs, although there were significant inverse associations of food responsiveness, enjoyment of food, and FAPP with 1,2,3,4,6,7,8,9-octachlorodibenzofuran, a PCDF isomer whose levels in breast milk were low (25.4% of samples were below the limit of detection; Table 1). Levels of 1,2,3,7,8,9-hexachlorodibenzofuran and 1,2,3,4,7,8,9-heptachlorodibenzofuran were also associated with enjoyment of food scores in boys (Table 3). Levels of 1,2,3,4,6,7,8,9-octachlorodibenzo-*p*-dioxin in boys were inversely associated with the fussiness score (β = − 0.225, *p* = 0.044), suggesting a connection between this dioxin congener and decreased food avoidance behavior.

**Table 3:** Associations between dioxin levels and Children’s Eating Behaviour Questionnaire scores in boys aged 3 years

	FR		EF		DD		EO		FAPP	
	Beta	(95% CI)	Beta	(95% CI)	Beta	(95% CI)	Beta	(95% CI)	Beta	(95% CI)
2,3,7,8-TetraCDD	0.063	(− 0.167, 0.292)	0.080	(− 0.154, 0.315)	0.047	(− 0.172, 0.266)	0.101	(− 0.126, 0.328)	0.101	(−0.125, 0.326)
1,2,3,7,8-PentaCDD	0.012	(− 0.204, 0.228)	− 0.156	(− 0.374, 0.063)	0.119	(− 0.085, 0.323)	0.061	(− 0.152, 0.274)	− 0.039	(− 0.250, 0.173)
1,2,3,4,7,8-HexaCDD	− 0.059	(− 0.288, 0.171)	− 0.186	(− 0.417, 0.046)	−0.006	(− 0.224, 0.213)	−0.002	(− 0.229, 0.225)	− 0.110	(− 0.335, 0.114)
1,2,3,6,7,8-HexaCDD	0.031	(− 0.183, 0.246)	−0.121	(− 0.339, 0.098)	0.012	(− 0.192, 0.217)	− 0.033	(− 0.179, 0.246)	−0.023	(− 0.234, 0.189)
1,2,3,7,8,9-HexaCDD	0.011	(− 0.194, 0.217)	−0.159	(− 0.366, 0.048)	0.092	(− 0.103, 0.287)	− 0.023	(− 0.226, 0.181)	−0.068	(− 0.269, 0.134)
1,2,3,4,6,7,8-HeptaCDD	0.024	(−0.247, 0.199)	− 0.049	(− 0.277, 0.180)	−0.006	(− 0.219, 0.207)	−0.151	(− 0.370, 0.068)	−0.082	(− 0.301, 0.137)
OctaCDD	0.036	(−0.178, 0.250)	−0.003	(− 0.221, 0.216)	−0.029	(− 0.233, 0.174)	−0.080	(− 0.291, 0.131)	−0.007	(− 0.217, 0.203)
2,3,7,8-TetraCDF	0.084	(−0.115, 0.283)	0.028	(−0.176, 0.231)	0.013	(−0.177, 0.203)	−0.044	(− 0.241, 0.153)	0.043	(− 0.152, 0.239)
1,2,3,7,8-PentaCDF	−0.008	(− 0.204, 0.189)	−0.118	(− 0.317, 0.081)	−0.003	(− 0.190, 0.184)	−0.124	(− 0.317, 0.069)	0.094	(− 0.286, 0.098)
2,3,4,7,8-PentaCDF	−0.030	(− 0.248, 0.189)	−0.152	(− 0.373, 0.069)	0.048	(− 0.159, 0.256)	0.028	(− 0.188, 0.244)	−0.071	(− 0.285, 0.143)
1,2,3,4,7,8-HexaCDF	−0.064	(− 0.263, 0.135)	−0.162	(− 0.363, 0.039)	− 0.007	(− 0.197, 0.182)	0.001	(− 0.198, 0.197)	− 0.103	(− 0.298, 0.091)
1,2,3,6,7,8-HexaCDF	− 0.067	(− 0.236, 0.163)	− 0.158	(− 0.359, 0.043)	0.014	(− 0.176, 0.204)	0.018	(− 0.180, 0.215)	− 0.081	(− 0.276, 0.115)
1,2,3,7,8,9-HexaCDF	0.053	(− 0.161, 0.266)	− 0.216	(− 0.431, − 0.002)*	0.015	(− 0.098, 0.308)	− 0.165	(− 0.374, 0.044)	− 0.115	(− 0.324, 0.094)
2,3,4,6,7,8-HexaCDF	− 0.158	(− 0.363, 0.048)	− 0.121	(− 0.332, 0.090)	− 0.013	(− 0.211, 0.185)	− 0.042	(− 0.248, 0.164)	−0.151	(− 0.353, 0.051)
1,2,3,4,6,7,8-HeptaCDF	−0.079	(− 0.275, 0.118)	−0.180	(− 0.378, 0.018)	−0.040	(− 0.228, 0.147)	−0.044	(− 0.239, 0.150)	− 0.133	(− 0.325, 0.059)
1,2,3,4,7,8,9-HeptaCDF	−0.090	(− 0.281, 0.100)	− 0.204	(− 0.395, − 0.010)*	−0.044	(− 0.226, 0.138)	− 0.081	(− 0.270, 0.108)	−0.161	(− 0.346, 0.024)
OctaCDF	−0.250	(− 0.434, − 0.066)**	−0.263	(− 0.451, − 0.080)**	−0.003	(− 0.185, 0.179)	− 0.151	(− 0.338, 0.035)	−0.296	(− 0.474, − 0.120)**
TEQ-PCDDs	0.029	(− 0.197, 0.255)	− 0.126	(− 0.356, 0.104)	0.096	(− 0.118, 0.311)	0.067	(− 0.156, 0.291)	− 0.015	(− 0.237, 0.207)
TEQ-PCDFs	− 0.045	(− 0.252, 0.161)	− 0.166	(− 0.375, 0.043)	0.017	(− 0.180, 0.214)	0.012	(− 0.193, 0.217)	− 0.091	(− 0.293, 0.112)
TEQ-PCDDs/Fs	− 0.010	(− 0.232, 0.212)	− 0.159	(− 0.384, 0.066)	0.063	(− 0.148, 0.274)	0.040	(− 0.179, 0.260)	−0.059	(− 0.277, 0.159)

Beta: Standardized regression coefficient controlling the following covariates: Location, maternal age, parity, maternal body mass index, maternal education, family income, gestational weeks at birth, and children’s age at the time of the survey

*CDD* chlorinated dibenzo-*p*-dioxin, *CDF* chlorinated dibenzofuran, *CI* confidence interval, *DD* desire to drink, *EF* enjoyment of food, *EO* emotional over-eating, *FAPP* food approach, *FR* food responsiveness, *PCDD* polychlorinated dibenzo-*p*-dioxin, *PCDF* polychlorinated dibenzofuran, *TEQ* toxic equivalency

The FAPP was calculated as FR + EF + DD + EO

*p < 0.05; **p < 0.01

**Table 4:** Associations between dioxin levels and Children’s Eating Behaviour Questionnaire scores in girls aged 3 years

	FR		EF		DD		EO		FAPP	
	Beta	(95% CI)	Beta	(95% CI)	Beta	(95% CI)	Beta	(95% CI)	Beta	(95% CI)
2,3,7,8-TetraCDD	− 0.220	(− 0.458, 0.017)	− 0.079	(− 0.327, 0.168)	− 0.012	(− 0.249, 0.224)	0.016	(− 0.230, 0.262)	−0.156	(− 0.394, 0.082)
1,2,3,7,8-PentaCDD	−0.131	(− 0.399, 0.138)	− 0.219	(− 0.490, 0.052)	−0.325	(− 0.576, − 0.074)*	−0.169	(− 0.439, 0.101)	−0.251	(− 0.512, 0.009)
1,2,3,4,7,8-HexaCDD	−0.002	(− 0.268, 0.263)	− 0.272	(− 0.535, − 0.010)*	−0.291	(− 0.539, − 0.043)*	−0.086	(− 0.353, 0.181)	− 0.166	(− 0.426, 0.093)
1,2,3,6,7,8-HexaCDD	0.011	(− 0.255, 0.277)	−0.246	(− 0.510, 0.019)	− 0.239	(− 0.491, 0.013)	−0.085	(− 0.353, 0.183)	−0.146	(− 0.407, 0.115)
1,2,3,7,8,9-HexaCDD	−0.036	(− 0.281, 0.210)	−0.293	(− 0.533, − 0.053)*	−0.311	(− 0.537, − 0.085)**	−0.078	(− 0.325, 0.170)	−0.192	(− 0.430, 0.047)
1,2,3,4,6,7,8-HeptaCDD	0.032	(−0.225, 0.289)	− 0.274	(− 0.528, − 0.021)*	−0.316	(− 0.554, − 0.079)*	−0.052	(− 0.312, 0.207)	−0.133	(− 0.385, 0.119)
OctaCDD	0.053	(−0.189, 0.295)	−0.074	(− 0.320, 0.172)	−0.188	(− 0.419, 0.043)	−0.025	(− 0.368, 0.118)	−0.059	(− 0.298, 0.180)
2,3,7,8-TetraCDF	−0.097	(− 0.351, 0.157)	−0.226	(− 0.480, 0.028)	−0.186	(− 0.429, 0.057)	0.178	(− 0.076, 0.432)	−0.083	(− 0.334, 0.169)
1,2,3,7,8-PentaCDF	0.170	(−0.064, 0.403)	−0.270	(− 0.503, − 0.037)*	−0.298	(− 0.517, − 0.080)*	0.190	(−0.045, 0.425)	0.055	(−0.179, 0.289)
2,3,4,7,8-PentaCDF	−0.059	(−0.327, 0.210)	− 0.276	(− 0.542, − 0.01)*	−0.280	(− 0.533, − 0.028)*	−0.084	(− 0.355, 0.187)	−0.200	(− 0.462, 0.061)
1,2,3,4,7,8-HexaCDF	0.011	(−0.245, 0.267)	−0.248	(− 0.502, 0.006)	−0.357	(− 0.590, − 0.124)**	−0.076	(− 0.334, 0.181)	−0.143	(− 0.394, 0.107)
1,2,3,6,7,8-HexaCDF	−0.007	(− 0.261, 0.246)	−0.280	(− 0.529, − 0.031)*	−0.331	(− 0.563, − 0.098)**	−0.107	(− 0.362, 0.148)	−0.182	(− 0.429, 0.064)
1,2,3,7,8,9-HexaCDF	0.012	(−0.238, 0.263)	−0.224	(− 0.474, 0.226)	−0.213	(− 0.441, 0.036)	−0.011	(− 0.264, 0.243)	−0.102	(− 0.349, 0.144)
2,3,4,6,7,8-HexaCDF	0.029	(−0.228, 0.286)	− 0.192	(− 0.450, 0.066)	−0.276	(− 0.516, − 0.035)*	−0.127	(− 0.385, 0.131)	−0.129	(− 0.381, 0.123)
1,2,3,4,6,7,8-HeptaCDF	0.069	(−0.177, 0.314)	−0.223	(− 0.468, 0.022)	−0.352	(− 0.575, − 0.128)**	−0.013	(− 0.262, 0.236)	−0.071	(− 0.314, 0.172)
1,2,3,4,7,8,9-HeptaCDF	0.076	(−0.175, 0.328)	−0.098	(− 0.354, 0.158)	−0.292	(− 0.527, − 0.058)*	0.039	(−0.216, 0.293)	0.015	(−0.235, 0.264)
OctaCDF	0.005	(−0.215, 0.224)	0.016	(−0.208, 0.240)	−0.204	(− 0.411, 0.004)	0.189	(− 0.028, 0.406)	0.094	(− 0.122, 0.309)
TEQ-PCDDs	−0.138	(− 0.402, 0.126)	−0.182	(− 0.450, 0.085)	−0.220	(− 0.473, 0.032)	−0.109	(− 0.376, 0.158)	−0.212	(− 0.470, 0.046)
TEQ-PCDFs	−0.012	(− 0.274, 0.249)	−0.276	(− 0.533, − 0.018)*	−0.331	(− 0.572, − 0.090)*	−0.080	(− 0.343, 0.183)	−0.171	(− 0.426, 0.084)
TEQ-PCDDs/Fs	−0.086	(− 0.351, 0.179)	−0.237	(− 0.502, 0.028)	−0.279	(− 0.528, − 0.030)*	−0.101	(− 0.368, 0.167)	−0.204	(− 0.463, 0.054)

Beta: Standardized regression coefficient controlling the following covariates: Location, maternal age, parity, maternal body mass index, maternal education, family income, gestational weeks at birth, and children’s age at the time of the survey

*CDD* chlorinated dibenzo-*p*-dioxin, *CDF* chlorinated dibenzofuran, *CI* confidence interval, *DD* desire to drink, *EF* enjoyment of food, *EO* emotional over-eating, *FAPP* food approach, *FR* food responsiveness, *PCDD* polychlorinated dibenzo-*p*-dioxin, *PCDF* polychlorinated dibenzofuran, *TEQ* toxic equivalency

The FAPP was calculated as FR + EF + DD + EO

*p < 0.05; **p < 0.01

In girls, we found significant inverse associations between levels of TEQ-PCDFs and enjoyment of food scores, and between levels of TEQ-PCDFs and TEQ-PCDDs/Fs and desire to drink scores. Among PCDF isomers, two pentachlorodibenzofuran isomers with high TEFs and 1,2,3,6,7,8-hexachlorodibenzofuran were associated with lower enjoyment of food scores, and seven PCDF isomers were associated with lower desire to drink scores in girls (Table 4). In addition, four PCDD isomers had an inverse association with desire to drink scores, contributing to an association between TEQ-PCDDs/Fs and decreased desire to drink scores (Table 4). FAPP was not significantly associated with any dioxin TEQs or isomers in girls. No significant association was found between dioxins and scores of food-avoidance behaviors such as satiety responsiveness, slowness in eating, fussiness, emotional under-eating, or FAVD in girls.

The findings suggested significant associations between exposure to dioxin, as indicated by TEQ-PCDF and/or TEQ-PCDD/F values, and enjoyment of food and desire to drink scores, with sex-specific differences. We therefore analyzed the effects of the two main factors, sex and dioxin type (low- and high-exposure groups; < and ≥ 75th percentile of TEQ-PCDF and TEQ-PCDD/F levels, respectively) on enjoyment of food and desire to drink scores using the general linear model after adjusting for covariates (Table 5). We found no significant effects of sex or TEQ-PCDD/F levels on enjoyment of food or desire to drink scores. TEQ-PCDF levels, however, were significantly associated with enjoyment of food scores (F[1173] = 6.983, *p* = 0.009), without a significant effect by sex. The adjusted mean enjoyment of food score was 3.8, significantly lower in the high-exposure TEQ-PCDF group than in the low-exposure group (4.1). The effect of TEQ-PCDFs on the desire to drink score was borderline significant (F[1173] = 2.927, *p* = 0.089), but interaction between sex and TEQ-PCDF levels was significant (F[1173] = 4.897, *P* = 0.028), with a lower adjusted mean in the high-exposure TEQ-PCDF group (2.8) than in the low-exposure group (3.5) in girls.

**Table 5:** Effects of sex and dioxin type on Children’s Eating Behaviour Questionnaire (CEBQ) scores

CEBQ	Main effects and interaction	df	Mean SS	F-value	*P*-value
EF	Sex (Girls/Boys)	1	0.292	0.312	0.577
	TEQ-PCDDs/Fs (High/Low)	1	2.649	2.832	0.094
	Sex * TEQ-PCDDs/Fs	1	0.163	0.175	0.676
	Sex (Girls/Boys)	1	0.056	0.061	0.806
	TEQ-PCDFs (High/Low)	1	6.388	6.983	0.009
	Sex * TEQ-PCDFs	1	0.007	0.007	0.932
DD	Sex (Girls/Boys)	1	1.397	1.664	0.199
	TEQ-PCDDs/Fs (High/Low)	1	2.047	2.438	0.120
	Sex * TEQ-PCDDs/Fs	1	2.047	2.438	0.120
	Sex (Girls/Boys)	1	1.917	2.325	0.129
	TEQ-PCDFs (High/Low)	1	2.413	2.927	0.089
	Sex * TEQ-PCDFs	1	4.037	4.897	0.028

The general linear model was adjusted for the following covariates: Location, maternal age, parity, maternal body mass index, maternal education, family income, gestational weeks at birth, and children’s age at the time of the survey

*DD* desire to drink, *df* degrees of freedom, *EF* enjoyment of food, *PCDD* polychlorinated dibenzo-*p*-dioxin, *PCDF* polychlorinated dibenzofuran, *SS* sum of squares, *TEQ* toxic equivalency

The cutoff value of TEQ-PCDFs for high- and low-exposure groups was 7.6 pg-TEQ/g lipid (75th percentile)

The cutoff value of TEQ-PCDDs/Fs for high- and low-exposure groups was 17.8 pg-TEQ/g lipid (75th percentile)

## Discussion

### Dioxin exposure and eating behavior in children aged 3 years

In the present study, high levels of TEQ-PCDFs and TEQ-PCDDs/Fs were associated with reduced food approach, but no association was found between eating behavior scores and levels of TCDD, the main congener in Agent Orange. In girls, high- and low-level exposures to TEQ-PCDDs/Fs were not associated with height, weight, or BMI (data not shown). BMI was not associated with CEBQ scores, and a decrease in positive eating score was not related to emaciation. In boys, while dioxin exposure was not related to eating behavior, BMI was positively associated with enjoyment of food scores and inversely correlated with satiety responsiveness scores, suggesting that eating behavior affects BMI.

In our previous study performed in the same cohort, we reported that perinatal exposure to TCDD was associated with an increase in autistic traits, particularly in boys [9]. Children with autism are known to eat a significantly narrower range of foods [11] and are more likely to exhibit fussy eating behavior [12] than typically developed children. The present findings suggest a possible alteration in eating behavior in boys with autistic traits exposed to high levels of TCDD, but we did not find an association between TCDD and CEBQ scores, although there were significant associations between the ASRS and slowness in eating and emotional under-eating scores. These unexpected findings may be explained by the report of Nadon and colleagues that children with autistic traits have fewer sensory processing problems than are frequently observed in autism spectrum disorder children with eating problems [25].

In the present study, TEQ-PCDFs of congeners with comparatively lower TEFs than PCDDs were associated with decreased CEBQ scores. Some high-chlorinated PCDD isomers with lower TEF values, such as heptachlorodibenzo-*p*-dioxin and octachlorodibenzo-*p*-dioxin, were associated with eating behaviors; this suggests that the mechanism altering eating behavior in these children may not be the toxicity of TCDD-like aryl hydrocarbon receptor agonists. A similar study in areas contaminated by PCDFs and high-chlorinated PCDD isomers in other countries than Vietnam would be required to validate the effects on eating behavior in young children.

### Neurotoxic effects of dioxin exposure on appetite and taste preference in animals

We conducted the present study to assess whether dioxin exposure might alter eating behaviors in children. Animal studies have linked perinatal dioxin exposure with poor neurodevelopment. Pretreatment with TCDD was reported to completely block the effects of lesions in the ventromedial hypothalamus of the rat brain, leading to hypophagia and weight loss, suggesting that TCDD exposure affects neurological control of appetite [13]. Food intake has been reported to be regulated by a neuronal network in the limbic system of the rat brain, and involvement of GABAergic neurons in this network has been suggested to control feeding responses, both excitation and suppression, via bidirectional interactions [26]. Because low doses of TCDD have been shown to influence development of GABAergic neurons in the rat brain [27], perinatal dioxin exposure may affect GABAergic neurons in the brain network that control food intake and appetite in humans as well.

Previous studies have suggested that perinatal dioxin exposure affects taste preference in rats, particular in females. Perinatal TCDD and dioxin-like PCB exposure altered the expression of saccharin preference behavior in adult female rats [28]. Nishijo and colleagues reported that female pups exposed to TCDD during the perinatal period preferred the bitter taste of histidine and lysine solutions using a choice paradigm of six amino acid solutions [19].

### Sex-specific effects in humans

Growth restriction has been reported in infants with Yusho disease whose mothers ingested rice oil contaminated with PCDDs/Fs and PCBs during pregnancy, and particularly in boys [29]. In our previous follow-up studies of the same birth cohort [6–10], we also found adverse health effects of perinatal dioxin exposure on weight, and height, and head and abdominal circumferences, and neurodevelopment that were more pronounced in boys. A study in Dutch children aged 6–8 reported endocrine-disrupting effects of perinatal exposure to PCDDs/Fs and PCBs manifesting as more feminized play behavior in boys [30], suggesting a greater susceptibility to dioxin in males. In the present study of eating behaviors, however, a significant influence of perinatal dioxin exposure was found in girls.

In children, eating behaviors have been targeted in preventing obesity and over-eating. One study reported that boys were more inclined to have a post-meal snack than girls, a habit leading to weight gain [31]. A study of elementary school children in Japan found that boys were more interested in food and preferred fatty foods, and that food interest scores were associated with energy density, fat energy content, and saturated fatty acid scores [32]. A cohort study of 2-year-old children born at preterm found that being female was associated with eating difficulties [33], while a study in 5-year-old American children concluded that parental pressure on girls to eat more was associated with the emergence of dietary restraint and emotional disinhibition, as well as with decreased responsiveness to internal hunger and satiety cues [34]. In addition, Nordin and colleagues found that in young Swedish adults, food rejection and aversion were more common in women [35]. These studies indicate that females are more inclined than males to develop aversion to foods, and further suggest that the 3-year-old girls in the present study may be susceptible to dioxin-induced effects on emotion-related food aversion. Longer follow-up periods would be required to elucidate the sex-specific effects of dioxin exposure on emotion-related eating and food preferences in this cohort.

### Sources of dioxin contamination and prevention of health effects

We found that TEQ-PCDFs contributed more to increased FAPP scores in girls than TEQ-PCDDs, including TCDD. Our previous study of dioxin exposure sources indicated that PCDF levels may increase with increased intake of marine food sources such as shrimp and with longer residency in contaminated areas [36]. Ongoing remediation of dioxin contamination in the former US airbases should be completed to decrease maternal dioxin body burdens leading to perinatal exposure.

## Conclusion

Perinatal exposure to dioxin affected eating behavior in 3-year-old children and particularly in girls. Future research should clarify the effects of dioxins on emotional development that affects eating styles and behaviors, leading to health problems such as emaciation or obesity in later life.

## Additional file

Additional file 1: Data of CEBQ and dioxins in 3-year-old Vietnamese children (a cross sectional study). (XLSX 43 kb)

## Abbreviations

ASRS: Autism Spectrum Rating Scales
CEBQ: Children’s Eating Behaviour Questionnaire
dl-PCBs: Dioxin-like polychlorinated biphenyls
FAPP: Food approach
FAVD: Food avoidance
PCDDs: Polychlorinated dibenzo-p-dioxins
PCDFs: Polychlorinated dibenzofurans
TCDD: 2,3,7,8-tetrachlorodibenzo-p-dioxin
TEF: Toxic equivalency factor
TEQ: Toxic equivalency
